# Supplementary material for: Modeling glioblastoma heterogeneity as a dynamic network of cell states
Source: Mol Syst Biol. 2021 Sep 16;17(9):e10105. doi: 10.15252/msb.202010105 (PMC8444284; doi:10.15252/msb.202010105)
Supplement: Supplementary file 6 — Source Data for Figure 5 [file MSB-17-e10105-s004.zip › Figure5A_sourcedata/GSEA_3071/hallmarks_stateIV.GseaPreranked.1623742551702/HALLMARK_ANGIOGENESIS.html]

Details for gene set HALLMARK\_ANGIOGENESIS[GSEA]

|  || Dataset | state53071 |
| Phenotype | NoPhenotypeAvailable |
| Upregulated in class | na\_neg |
| GeneSet | HALLMARK\_ANGIOGENESIS |
| Enrichment Score (ES) | -0.59546626 |
| Normalized Enrichment Score (NES) | -2.148045 |
| Nominal p-value | 0.0 |
| FDR q-value | 2.7703083E-4 |
| FWER p-Value | 0.002 |
Table: GSEA Results Summary

  

Fig 1: Enrichment plot: HALLMARK\_ANGIOGENESIS      
 Profile of the Running ES Score & Positions of GeneSet Members on the Rank Ordered List

  

| PROBE | GENE SYMBOL | GENE\_TITLE | RANK IN GENE LIST | RANK METRIC SCORE | RUNNING ES | CORE ENRICHMENT || 1 | STC1 |  |  | 97 | 1.547 | 0.0419 | No |
| 2 | APP |  |  | 136 | 1.426 | 0.0904 | No |
| 3 | TNFRSF21 |  |  | 232 | 1.184 | 0.1185 | No |
| 4 | FGFR1 |  |  | 602 | 0.726 | 0.0768 | No |
| 5 | VEGFA |  |  | 1010 | 0.518 | 0.0198 | No |
| 6 | PTK2 |  |  | 1631 | 0.362 | -0.0837 | No |
| 7 | KCNJ8 |  |  | 1933 | 0.308 | -0.1288 | No |
| 8 | MSX1 |  |  | 2021 | 0.293 | -0.1339 | No |
| 9 | JAG1 |  |  | 4168 | -0.559 | -0.5193 | No |
| 10 | ITGAV |  |  | 4312 | -0.601 | -0.5230 | No |
| 11 | FSTL1 |  |  | 4446 | -0.653 | -0.5228 | No |
| 12 | VCAN |  |  | 4464 | -0.661 | -0.5002 | No |
| 13 | PDGFA |  |  | 4967 | -1.039 | -0.5549 | Yes |
| 14 | TIMP1 |  |  | 5086 | -1.257 | -0.5283 | Yes |
| 15 | COL5A2 |  |  | 5097 | -1.282 | -0.4802 | Yes |
| 16 | JAG2 |  |  | 5143 | -1.394 | -0.4343 | Yes |
| 17 | NRP1 |  |  | 5177 | -1.553 | -0.3800 | Yes |
| 18 | S100A4 |  |  | 5189 | -1.605 | -0.3195 | Yes |
| 19 | LUM |  |  | 5271 | -2.487 | -0.2378 | Yes |
| 20 | SPP1 |  |  | 5277 | -2.671 | -0.1346 | Yes |
| 21 | COL3A1 |  |  | 5287 | -3.508 | 0.0006 | Yes |
Table: GSEA details [plain text format]

  

Fig 2: HALLMARK\_ANGIOGENESIS: Random ES distribution      
 Gene set null distribution of ES for **HALLMARK\_ANGIOGENESIS**
